# Effects of High-Intensity Training on Complete Blood Count, Iron Metabolism, Lipid Profile, Liver, and Kidney Function Tests of Professional Water Polo Players

**DOI:** 10.3390/diagnostics14182014

**Published:** 2024-09-11

**Authors:** Frane Bukvić, Domagoj Marijančević, Helena Čičak, Ana-Maria Šimundić, Daria Pašalić, Lora Dukić

**Affiliations:** 1 Department of Orthopedics and Trauma Surgery, University Hospital ‘Sveti Duh’, 10000 Zagreb, Croatia; frane.bukvic@gmail.com; 2 Department of Clinical Chemistry, University Hospital Centre ‘Sestre Milosrdnice’, 10000 Zagreb, Croatia; domagoj.marijancevic@kbcsm.hr; 3 Department of Medical Laboratory Diagnostics, University Hospital ‘Sveti Duh’, 10000 Zagreb, Croatia; helena.cicak5@gmail.com; 4 Unit for Preanalytics, Department of Global Medical & Clinical Affairs, Business Greiner Bio-One GmbH, 4550 Kremsmünster, Austria; am.simundic@gmail.com; 5 Faculty of Pharmacy and Medical Biochemistry, Zagreb University, 10000 Zagreb, Croatia; 6 Department of Medical Chemistry, Biochemistry and Clinical Chemistry, University of Zagreb School of Medicine, 10000 Zagreb, Croatia; 7 Clinical Department of Laboratory Diagnostics, Clinical Hospital Center Rijeka, 51000 Rijeka, Croatia; lora.dukic@gmail.com

**Keywords:** biochemical markers, CBC, high-intensity training, kidney function tests, lipid profile, liver function tests, water polo players

## Abstract

AIM: Our goal was to examine the effect of high-intensity physical activity on changes in the lipid profile, complete blood count (CBC), iron metabolism, and kidney and liver function tests of professional water polo players. METHODS: This study included twenty professional male water polo players. Blood sampling was carried out at the beginning of the season and during periods of high-intensity training. CBCs were determined with a Siemens Advia 2120i hematology analyzer. A Beckman CoulterAU680 chemistry analyzer was used to determine the serum concentrations/activities of lipid profiles and liver and kidney function test analytes. The lipid athlete scores were also determined. RESULTS: The mean corpuscular volume (*p* = 0.006), platelet count (*p* = 0.008), and mean platelet volume (*p* < 0.001) significantly decreased during the high-intensity period, compared with the beginning of the season. The total iron-binding capacity increased (*p* = 0.001), and ferritin concentrations significantly declined (*p* = 0.017). The lipid profiles revealed a significant difference between phases, with slight increases in serum total (*p* = 0.025) and LDL cholesterol (*p* = 0.002) levels and a decrease in triglyceride concentrations (*p* = 0.040) in the high-intensity period. During the high-intensity period, the liver and kidney function tests showed a substantial positive effect on lactate dehydrogenase levels (*p* < 0.001), aspartate aminotransferase (*p* = 0.028) serum activity, and total protein concentrations (*p* = 0.033), compared with the beginning of the season. CONCLUSIONS: Water polo players might exhibit a decrease in some CBC parameters, an increase in LDL cholesterol, and a decrease in liver function biomarkers due to intense training at the peak of the competitive season. Kidney function biomarkers remain unchanged.

## 1. Introduction

Frequent physical activity reduces the risk of various illnesses, including coronary artery disease, stroke, diabetes, and malignant disorders, and it has been stated that “It helps prevent high blood pressure and obesity while promoting mental health, overall quality of life, and well-being” [1]. Competitive sports emphasize achievement and skill development via rigorous training, while recreational sports focus on participation, enjoyment, and social interaction with lower stress and commitment levels [2]. However, one can get hurt, burn out, or develop long-term health problems. Laboratory testing can assist in tracking these benefits and drawbacks, alongside carefully planned training and conditioning programs and other lifestyle elements that are critical to athletes’ health.

Sports laboratory medicine is a preventive discipline focused on assessing an athlete’s health and performance status condition [3]. Intense and continuous physical training and competition can alter serum concentrations of various laboratory markers [4]. A specific subset of laboratory parameters is intended to evaluate an athlete’s health and thereby determine appropriate treatment actions to optimize athletic performance while reducing the risk of injury [3]. It is well known that certain conditions, especially those related to cardiovascular diseases (CVDs) or metabolic disorders, can go unnoticed, particularly without a previous history or distinctive signs, potentially leading to severe incidents or even death [5].

An athlete’s health can be evaluated with a complete blood count (CBC) [6]. The shape and number of red blood cells (RBCs), as well as the concentration of hemoglobin in the blood, can indicate if an athlete’s muscles are receiving adequate oxygen. A lower hemoglobin concentration or irregularly formed RBCs are signs of anemia [7]. Athletes trained in endurance sports may develop sports anemia. Physical activity can induce erythropoiesis as well as reduce RBC mass via intravascular hemolysis, which is triggered by mechanical rupture when red blood cells pass through capillaries in contracting muscles and the compression of red cells [8]. Acute changes in training load (5 days before blood collection) altered the hematological module of Athlete Biological Passport (ABP) parameters [e.g., Hb] via changes in plasma volume; conversely, persistent variations in training load (for more than a month) did not [9]. Additionally, the same study noted considerable variations in plasma volume over time. Iron deficiency, particularly in endurance athletes, is common in sports and usually manifests as iron deficiency without anemia (IDNA). Athletes are predisposed to iron shortage due to various factors, including increased iron losses during exercise caused by micro-ischemia, hemolysis, and perspiration [10]. A protocol for optimizing athletes’ iron status may improve their physical performance [10].

A carefully chosen aerobic exercise program positively affects various biochemical variables of the liver in healthy adult male athletes. After 12 weeks of aerobic training, adult athletes’ liver function appeared to improve [11]. Determining a liver enzyme profile to evaluate the metabolic response to aerobic exercise could be an early indicator of training-related damage. The activities of gamma-glutamyl transferase (GGT), alanine aminotransferase (ALT), and aspartate aminotransferase (AST) were found to be potentially useful for assessing metabolic processes in high-performance athletes [12].

Soccer-related exercise throughout the season can lead to renal function changes that minimize dehydration through increasing the reabsorption of urea and conservation of water [13]. The serum creatinine concentration may appear pathological in some athletes. The interpretation of these data should consider the athlete’s status [14]. Blood creatinine concentrations in athletes are higher than those in sedentary adults. Nevertheless, the population of athletes is not homogeneous, and disparities can be seen amongst athletes competing in various sports disciplines. Creatinine concentrations fluctuate during the competitive season in several sports, with higher concentrations indicating more rigorous exertion [14].

It is generally accepted that regular physical activity reduces the risk of cardiovascular disease and incident situations and improves the lipid profile of physically active persons. The discovery of dyslipidemia in athletes has generated interest in developing preventive methods to reduce cardiovascular events. However, although professional athletes have favorable lipid profiles, several studies have shown the significance of monitoring continuous lipid profiles in athletes to detect cardiovascular risk factors proactively. Specifically, previous investigations found dyslipidemia in more than 20% of professional athletes (such as skiers and bikers) [15].

The performances of professional athletes are monitored by analyzing blood biomarkers and fitness indicators [16]. Changes in blood biomarkers may be necessary for preventing overtraining and potential injuries and maintaining overall health during the intense efforts of the competitive season in many sports such as water polo. The scientific literature mostly presents results on the influence of physical activity on laboratory test results in shorter periods, while data on the cumulative effect during the competitive season are relatively scarce.

We assumed that the lipid profile, CBC, iron metabolism, and kidney and liver function tests of professional water polo players would change during high-intensity training compared with the beginning of the competitive season, mostly expecting favorable effects. We aimed to examine the effect of high-intensity physical activity on changes in the lipid profile, CBC, iron metabolism, and kidney and liver function tests of professional water polo players.

## 2. Materials and Methods

### 2.1. Study Participants

This retrospective and observational study included 20 professional male water polo players from Croatia ranging in age from 15 to 31, with a median age of 21. The experiments used a within-subjects design with repeated measures. The study group was highly homogeneous concerning lifestyle, sleeping patterns, dietary habits, and exercise modalities (i.e., cardio and strength). Information about a selection of the subjects has been provided in a previous publication [16]. The Ethical Committee at the University Hospital Sveti Duh in Zagreb, Croatia, approved this study in 2020. It was carried out per the Helsinki Declaration.

### 2.2. Blood Sampling

The experimental procedures in this study were conducted at the start of the season and during the high-intensity training period. The beginning of the season started with preparations for the new competitive season among the professional water polo players, which was preceded by a two-month vacation. During the two-month vacation, physical activity was minimized and included swimming and certain strength exercises to maintain fitness. The period of high-intensity training was the time between the middle and end of the competitive season when the largest number of competitive matches were played and the training intensity was highest, which led to the greatest fatigue in the players. The time between the two periods was 5.5 to 6 months. Blood sampling was performed after an overnight fast. Individuals were sitting and resting for 15 min before sampling. Blood collection was performed simultaneously during each phase of the investigation to minimize daily analyte variations. Blood was drawn from the antecubital vein before physical examinations into 3 mL whole blood tubes with EDTA and 10 mL serum tubes (Becton Dickinson, Franklin Lakes, NJ, USA) containing a clot activator. The serum collection tubes were held at room temperature for 30 min before being centrifuged at 3000× *g* for 10 min to allow for clotting.

### 2.3. Methods

CBCs were determined with a Siemens Advia 2120i 6-part differential hematology analyzer (Siemens Healtheneers, Erlangen, Germany).

A Beckman Coulter AU680 biochemistry analyzer (Beckman Coulter, Brea, CA, USA) was used to determine the serum parameters as follows: triglycerides (the GPO-PAP method), total cholesterol (CHOD-PAP), HDL cholesterol (the homogeneous enzyme immunoinhibition method), LDL cholesterol (the homogeneous enzyme method, CHO-PAP), AST and ALT (the photometric method, according to IFCC recommendations), lactate dehydrogenase (LD) (the kinetic UV test), bilirubin (the photometric method with stabilized diazonium salt), proteins (the photometric method), albumin (the photometric method with bromocresol green), creatinine (Jaffe’s kinetic method), urea (the kinetic UV test), iron, unsaturated iron-binding capacity (UIBC) (the photometric method), total iron-binding capacity (TIBC) (calculation), and ferritin and transferrin (the immunoturbidimetric method).

Lipid athlete scores (LASs) were determined according to the major score criteria, according to recently published literature data (Di Gioia et al., 2023) [15]. The major score included two criteria: LDL ≥ 2.98 mmol/L (115 mg/dL) and an LDL/HDL ratio ≥ 1.90. According to the criteria satisfied, participants were categorized as “O”—none were satisfied, “1”—1 was satisfied, or “2”—both were satisfied.

### 2.4. Statistics

The data were analyzed using Statistica (TIBCO Software Inc. 2020 Santa Clara, CA 95054, USA) data Science Workbench, version 14.0.0.15). The Shapiro–Wilk test was applied to determine whether the distribution was normal. The data were presented as medians, with interquartile ranges (IQRs) and non-parametric statistics applied, because there were fewer than 30 patients and due to deviations from a normal distribution. The paired Wilcoxon test was used for comparison to determine the differences between repeated measurement data. *p*-values of ≤ 0.05 were considered statistically significant.

## 3. Results

Table 1 shows the results of comparing the CBC parameters in the same subject group measured at the beginning of the season and during high-intensity exercise. The mean corpuscular volume (MCV), platelet count (PTC), and mean platelet volume (MPV) all significantly decreased during the high-intensity period.

Table 2 compares the iron serum concentrations, iron-binding capacity (total and unsaturated (TIBC and UIBC)), and iron carrier serum concentrations across the two seasons. During the high-intensity period, the TIBC considerably increased, while ferritin concentrations significantly decreased.

Table 3 compares the lipid profiles from the two periods. The results reveal a statistically significant difference between phases, with a modest increase in serum total and LDL cholesterol levels and a decrease in triglyceride concentrations in the high-intensity phase.

During the high-intensity period, the liver and kidney function tests showed a substantial positive effect on LD and AST serum activity and total protein concentrations (*p* = 0.033) compared with the beginning of the season (Table 4).

## 4. Discussion

Our comprehensive research, which spanned the entire competitive season, was aimed at understanding the impact of high-intensity training on professional athletes’ health. We expected to see a positive influence; however, our findings revealed different results. When we compared the beginning of the season to a period of high-intensity training and competition, we found some significant alterations in CBCs, iron metabolism, lipid profiles, and several liver function tests.

Regarding CBCs, our results show decreases in the MCV, PTC, and MPV. Additionally, a lower serum ferritin concentration and higher TIBC were observed. This indicates that professional water polo players might develop signs of anemia. Several studies that examined the influence of physical activity on changes in the results of CBCs and iron metabolism tests during the competitive season showed partially similar results. A study on male and female college athletes at their annual health screenings found that both sexes had decreased iron concentrations, but only females had full-fledged anemia. The WBC and differential blood count components of the CBC showed no clinical significance in either sex [6]. A review paper that collected data about the effect of professional sports on CBC parameters showed that throughout the season, hemoglobin and hematocrit levels in athletes from various sporting disciplines decrease during more strenuous training periods [7]. There was a substantial decrease and difference in CBC values but no clinically meaningful change in monocytes, red blood cells (RBCs), hemoglobin, or hematocrit after recreational SCUBA diving (after once-only exercise) [17]. Our study results show decreased PTC and MPV values in water polo players during the high-intensity training period compared with the beginning of the season. Contrary to our results, which show cumulative seasonal effects, the study compared MPV and PTC values before and after running (after once-only exercise) and showed a significant increase in both the PTC and MPV [18]. The close association between the baseline MPV and running time suggests that hyperactive platelets may have diverse effects on the endurance performance of half-marathon runners. A study of over 3000 Spanish professional athletes found that training influences the baseline ranges of biochemical and hematological parameters. Regarding hematological parameters, the upper limits for the hemoglobin MCV and MCH were significantly lower than those in the general population. Additionally, the interval of basal values for RBCs, hematocrit, and MCHC were like those described for the general population [19]. This indicates that the reference intervals should be defined separately and corrected accordingly for professional athletes. Simultaneously, the duration of physical activity in terms of professional athletes’ hard training should be given special consideration. It is well understood that once-only exercise disrupts cell homeostasis, resulting in inflammation, but recurrent physical activity improves immune function [20].

In terms of iron metabolism, transport, and storage, it is well known that iron deficiency without anemia is identified when ferritin levels are low, but hemoglobin levels are normal. The World Health Organization states that low ferritin levels lead to low hemoglobin levels, the hallmark of iron deficiency anemia. The reference interval for male adults’ ferritin levels (immunoturbidimetric method) is 20 to 250 mg/L. There is significant debate regarding the optimal ferritin concentration for athletes [10,21]. Investigations on iron metabolism in different sports in different groups showed decreased iron concentrations and ferritin levels and increased TIBC values [22]. The increased demand for iron during exercise and probable dietary iron insufficiencies, particularly among endurance athletes, enhance the risk of iron insufficiency. Furthermore, prolonged exercise can affect athletes’ iron absorption, use, storage, and total concentrations. Contrarily, iron excess may initially contribute to improved performance [23]. It is necessary to consider that even low-volume blood withdrawal can slightly affect transferrin and ferritin concentrations [24].

Although not clinically significant, the changes in the lipid profiles in our study exhibit a statistically significant increase in total and LDL cholesterol levels during the season of high-intensity training and competition compared with the beginning of the season. These findings are consistent with previously published research on 1058 Olympic athletes, which revealed that dyslipidemia is one of the most common cardiovascular (CV) risk factors in elite athletes [25]. Ultra-endurance athletes who followed a very low-carbohydrate/high-fat diet for over a year had distinct cholesterol profiles, including continuously higher LDL-C and HDL-C levels, fewer small LDL particles, and lipoprotein profiles consistent with increased insulin sensitivity [26]. The most common cause of sudden cardiac death (SDC) in older athletes is coronary artery disease. As a result, excessive cholesterol concentrations may lead to SDC among athletes [27]. Usually, consistent aerobic activity raises HDL cholesterol levels while decreasing LDL cholesterol levels. These effects vary based on a person’s health, nutrition, and level of physical activity. However, high-intensity training paired with a high-fat ketogenic diet may yield varied results. Therefore, endurance athletes can exhibit high cholesterol concentrations. It is a well-established fact that sports participation, whether amateur or professional, reduces the risk of cardiovascular disease [27]. However, athletes must remain vigilant and monitor risk factors such as lipid profiles to protect against unforeseen events because genetics, lifestyle, and nutrition can still significantly impact CV risk, even in athletes. Nonetheless, the following overarching principle remains: sports are a powerful tool in the fight against cardiovascular disease.

Our study on liver function tests during high-intensity training and competition revealed significant changes. We observed a significant decrease in serum LD and AST activity and a corresponding increase in protein concentrations as the season progressed from its beginning to its peak, i.e., the period of high-intensity training and competition. Serum bilirubin, albumin concentrations, and ALT activity remained almost unchanged, highlighting dynamic changes in liver function during the season. The diverse literature data for similar analyses across various sports suggests the potential influence of high-intensity physical activity on LFTs. This finding could significantly impact the work in understanding athlete health. After 12 weeks of aerobic training, a study involving 30 male football players reported a substantial reduction in bilirubin and globulin concentrations, but no significant change in alkaline phosphate, AST, ALT, total protein, or albumin levels [11]. This showed that adult athletes’ liver function could be improved with a 12-week aerobic exercise program. A study on 16 male teenage athletes also found that extended and consistent training sessions beneficially affect athletes’ liver enzymes [28]. In contrast, a study that involved weightlifting and was undertaken over a shorter period showed that liver function tests significantly increased for at least seven days immediately after weightlifting [29]. In that study, bilirubin GGT and ALP levels remained within the normal range, but AST, ALT, and LD levels increased. Thus, it is imperative to set appropriate limits for intense muscle training before and during clinical studies, especially when examining the cumulative impact of prolonged exercise. LFT determinations carried out immediately after physical activity can temporarily increase the results of these measurements due to the significant expression of these enzymes, such as AST and LD, observed in muscle tissue. Sporting activities, however, have a long-term positive impact on LFTs. Therefore, it is imperative to ensure that these tests are carried out after a precisely scheduled rest period and no intense physical activity.

Our results show that kidney function tests remain the same during high-intensity training and competition. Contrary to our results, a study performed on Brazilian soccer players showed that the clearance of creatinine, proteins, and albumin increases in the post-season period, while urea clearance decreases [13]. A study of seven healthy athletes that examined renal function at rest and after each workout in a half-triathlon race found that the glomerular filtration rate (represented by creatinine clearance) remained constant after each exercise event, while the tubular reabsorption rate (reported as urea clearance) decreased after swimming, cycling, and running compared with pre-exercise levels [30]. It is necessary to consider that certain groups of athletes consume a high-protein diet to a greater or lesser extent, which has dose-dependent structural and functional effects on the kidneys because of the increase in the glomerular filtration rate (eGFR) and serum urea concentrations [31]. It is well established that eating more high-quality proteins before and after exercise improves the adaptive response, increases glycogen and protein synthesis, and decreases breakdown. As a result, the International Society for Sports Nutrition recommends a safe and appropriate protein consumption of 1.4–2.0 g/kg/day because many studies have demonstrated that a high protein intake over a year does not alter lipids and liver function. Furthermore, athletes, including amateurs, consume more protein than is suggested over a more extended period, which can significantly impact kidney function [32].

Our study group was highly homogeneous in terms of dietary, lifestyle, and training habits. According to our knowledge and literature review, no study of a similar design has examined different blood parameters during the competitive season in professional water polo players. As a result, the studies used for comparison primarily included other sports with a similar timing of blood sampling. This study had certain limitations because we did not collect anthropometric data, which may have influenced the laboratory test results. BMI could affect lipid profiles and liver and kidney function tests. Other limitations included the small number of participants and the inclusion of other confounders related to lifestyle habits that might affect laboratory tests. Hence, future investigations should include more data about different confounders for the study participants. Additionally, we could expand this investigation by including a control group of participants who practice sports only recreationally or with those who do not practice sports at all to compare potential biases between two or more phases during the competitive season.

Blood biomarker monitoring in sports depends on several factors, including the right choice of biomarkers, testing schedule, effective interdisciplinary teamwork, suitable longitudinal statistical techniques, and many pre-analytic factors. The investigated relationships may contribute to improving the tracking of athletes’ performance, diet, and overall health.

## 5. Conclusions

Compared with the beginning of the season, hematological parameters showed a statistically significant but slight shift toward values that lead to anemia during the high-intensity period. However, this shift was not clinically significant. Additionally, water polo players might exhibit an increase in LDL cholesterol levels and a decrease in liver function biomarkers, while kidney function biomarkers remain unchanged. Additionally, active athletes need to track these risk variables to prevent the possibility of sudden incident events and establish lifestyle and physical activity habits.

## Figures and Tables

**Table 1:** Comparison of CBC results at the beginning of the season and during the high-intensity season.

		Beginning of the Season	High-Intensity Season	
	Reference Range	Median (25th; 75th)		*p*-Value
WBCs (×10^9^/L)	3.4–9.7	10.1 (8.8; 11.5)	9.8 (7.3; 11.7)	0.601
Neutrophils (%)	44–77	59.8 (48.9; 66.1)	67.1 (54.7; 78.2)	0.126
Lymphocytes (%)	20–46	28.3 (22.7; 35.8)	23.1 (15.1; 29.85)	0.126
Monocytes (%)	2 to 12	5.7 (4.75; 6.5)	5.0 (4.3; 6.4)	0.494
Eosinophils (%)	Up to 7	2.7 (1.7; 4.5)	1.95 (1.45; 2.6)	0.103
Basophils (%)	Up to 1	0.60 (0.50; 0.75)	0.55 (0.45; 0.90)	0.962
RBCs (×10^12^/L)	4.34–5.72	4.9 (4.8; 5.1)	5.1 (4.9; 5.3)	0.145
Hemoglobin (g/L)	138–175	148 (142; 153)	149 (146; 152)	0.587
Hematocrit (L/L)	0.415–0.530	0.44 (0.42; 0.45)	0.44 (0.42; 0.45)	0.881
MCV (fL)	83.0–97.2	87.8 (84.6; 89.8)	87.3 (84.5; 88.7)	0.006
MCH (pg)	27.4–33.9	30.1 (28.9; 30.6)	29.6 (28.8; 30.4)	0.108
RDW (%)	9–15	13.4 (13.2; 13.7)	13.3 (13.0; 13.5)	0.087
PTC(×10^9^/L)	158–424	281 (225; 314)	234 (214; 302)	0.008
MPV (fL)	6.8–10.4	7.7 (7.5; 8.1)	7.0 (6.6; 7.6)	<0.001

WBCs—white blood cells; RBCs—red blood cells; MCV—mean corpuscular volume; MCH—mean corpuscular hemoglobin; RDW—red cell distribution width; PTC—platelet count; MPV—mean platelet volume.

**Table 2:** Comparison of iron distribution parameters at the beginning of the season and during the high-intensity season.

		Beginning of the Season	High-Intensity Season	
	Reference Range	Median (25th; 75th)		*p*-Value
Fe (µmol/L)	7–33	11.3 (9.6; 15.89)	14.3 (11.3; 19.3)	0.062
UIBC (µmol/L)	28–68	47.3 (44.7; 52.1)	48.9 (45.2; 52.5)	0.279
TIBC (µmol/L)	50–81	60.1 (57.7; 63.9)	64.6 (62.2; 66.5)	0.001
Transferrin (g/L)	2.0–3.6	2.65 (2.55; 2.85)	2.75 (2.65; 2.80)	0.171
Ferritin (µg/L)	20–250	61.5 (50.0; 95.0)	60 (41.0; 85.0)	0.017

Fe—iron; TIBC—total iron-binding capacity; UIBC—unsaturated iron-binding capacity.

**Table 3:** Comparison of lipid profiles at the beginning of the season and during the high-intensity season.

	Reference Range	Beginning of the Season	High-Intensity Season	
		Median (25th; 75th)		*p*-Value
TC, mmol/L	<5	3.67 (3.38; 4.64)	3.77 (3.61; 4.62)	0.025
HDL-C, mmol/L	>1.2	1.21 (1.06; 1.24)	1.21 (1.15; 1.31)	0.023
LDL-C, mmol/L	<3	2.21 (1.93; 2.63)	2.42 (2.27; 3.14)	0.002
TAGs, mmol/L	<1.7	1.30 (0.84; 1.98)	0.97 (0.82; 1.21)	0.04
LDL-C/HDL-C		1.92 (1.74; 2.51)	2.10 (1.86; 2.45)	0.067
		N (ratio)		
LAS 0	0	10 (0.50)	5 (0.25)	0.233
LAS 1		7 (0.35)	9 (0.45)	
LAS 2		3 (0.15)	6 (0.30)	

TC—total cholesterol; HDL-C—HDL cholesterol; LDL-C—LDL cholesterol; TAGs—triglycerides; LAS—Lipid Athlete Score [16].

**Table 4:** Kidney and liver function tests at the beginning of the season and during the high-intensity season.

		Beginning of the Season	High-Intensity Season	
	Reference Range	Median (25th; 75th)		*p*-Value
LD, U/L	127–231	224 (204; 250)	195 (164; 208)	<0.001
AST, U/L	11–38	30.5 (26.0; 38.0)	27.0 (23.0; 33.0)	0.028
ALT, U/L	12–48	22.0 (19; 29)	22.0 (18.0; 29.0)	0.546
Bilirubin, µmol/L	3–20	8.35 (7.15; 9.60)	8.20 (7.17; 11.60)	0.888
Protein, g/L	66–81	76.0 (75; 78.5)	77.5 (77.0; 80.5)	0.033
Albumin, g/L	41–51	49.0 (47.0; 50.0)	48.5 (47.0; 50.0)	0.875
Urea, mmol/L	2.8–8.3	7.05 (6.25; 7.50)	6.95 (6.20; 8.05)	0.737
Creatinine, µmol/L	79–125	111 (106; 121)	114 (101; 127)	0.614
Creatinine clearance mL/s	1.5–2.3	1.87 (1.57; 2.17)	1.9 (1.50; 2.35)	0.627

LD—lactate dehydrogenase; AST—aspartate aminotransferase; ALT—alanine aminotransferase.

## Data Availability

The data supporting the findings of this study are available upon request from the corresponding author. The data are not publicly available due to privacy or ethical restrictions.
