# Correction: Bukvić et al. Effects of High-Intensity Training on Complete Blood Count, Iron Metabolism, Lipid Profile, Liver, and Kidney Function Tests of Professional Water Polo Players. *Diagnostics* 2024, *14*, 2014

**DOI:** 10.3390/diagnostics16111721

**Published:** 2026-06-03

**Authors:** Frane Bukvić, Domagoj Marijančević, Helena Čičak, Ana-Maria Šimundić, Daria Pašalić, Lora Dukić

**Affiliations:** 1 Department of Orthopedics and Trauma Surgery, University Hospital ‘Sveti Duh’, 10000 Zagreb, Croatia; frane.bukvic@gmail.com; 2 Department of Clinical Chemistry, University Hospital Centre ‘Sestre Milosrdnice’, 10000 Zagreb, Croatia; domagoj.marijancevic@kbcsm.hr; 3 Department of Medical Laboratory Diagnostics, University Hospital ‘Sveti Duh’, 10000 Zagreb, Croatia; helena.cicak5@gmail.com; 4 Unit for Preanalytics, Department of Global Medical & Clinical Affairs, Business Greiner Bio-One GmbH, 4550 Kremsmünster, Austria; am.simundic@gmail.com; 5 Faculty of Pharmacy and Medical Biochemistry, Zagreb University, 10000 Zagreb, Croatia; 6 Department of Medical Chemistry, Biochemistry and Clinical Chemistry, University of Zagreb School of Medicine, 10000 Zagreb, Croatia; 7 Clinical Department of Laboratory Diagnostics, Clinical Hospital Center Rijeka, 51000 Rijeka, Croatia; lora.dukic@gmail.com

There was an error in the original publication [1] regarding the age range of participants.

A correction has been made to the Materials and Methods, Study Participants, paragraph 1:

This retrospective and observational study included 20 professional male water polo players from Croatia ranging in age from 15 to 31, with a median age of 21.

A correction has been made to Inform Consent Statement:

Informed consent was obtained from all subjects and their guardians involved in the study.

The authors state that the scientific conclusions are unaffected. This correction was approved by the Academic Editor. The original publication has also been updated.
